# Tanshinone II A attenuates vascular remodeling through klf4 mediated smooth muscle cell phenotypic switching

**DOI:** 10.1038/s41598-020-70887-1

**Published:** 2020-08-17

**Authors:** Guanhua Lou, Wangming Hu, Ziqiang Wu, Huan Xu, Huan Yao, Yang Wang, Qinwan Huang, Baojia Wang, Li Wen, Daoying Gong, Xiongbing Chen, Yaping Shi, Lan Yang, Yiming Xu, Yong Wang

**Affiliations:** 1 Chengdu University of Traditional Chinese Medicine, College of Basic Medicine, Chengdu, China; 2 Chengdu University of Traditional Chinese Medicine, College Pharmacy, Chengdu, China; 3 Chengdu University of Traditional Chinese Medicine, Hospital of Chengdu University of Traditional Chinese Medicine, Chengdu, China; 4 Guangzhou Medical University, School of Basic Medical Sciences, Guangzhou, China

**Keywords:** Drug discovery, Molecular biology, Cardiology, Medical research, Molecular medicine

## Abstract

The aim of this study is to investigate the therapeutic role of Tanshinone II A, a key integrant from salvia miltiorrhiza, against pathological vascular remodeling. Completed ligation of mouse left common carotid arteries animal model and rat smooth muscle cells used to investigate the role of Tanshinone II A in regulating pathological vascular remodeling through hematoxylin and eosin staining, immunohistochemistry staining, immunofluorescence staining, adenovirus infection, real time PCR and western blotting. Our data demonstrated that Tanshinone II A treatment suppresses vascular injury-induced neointima formation. In vitro studies on rat smooth muscle cell indicated that Tanshinone II A treatment attenuates PDGF-BB induced cell growth, and promotes smooth muscle cell differentiated marker genes expression that induced by rapamycin treatment. Tanshinone II A treatment significant inhibits rat smooth muscle cell proliferation and migration. Tanshinone II A promotes KLF4 expression during smooth muscle phenotypic switching. Overexpression of KLF4 exacerbates Tanshinone II A mediated smooth muscle cell growth inhibition. Tanshinone II A plays a pivotal role in regulating pathological vascular remodeling through KLF4 mediated smooth muscle cell phenotypic switching. This study demonstrated that Tanshinone II A is a potential therapeutic agent for vascular diseases.

## Introduction

Vascular smooth muscle cells phenotypic switching contributes to development of variety vascular diseases, including post angioplasty restenosis, aneurysm, atherosclerosis and pulmonary hypertension^1,2^. Maturated smooth muscle cell exhibits dramatically plasticity. The principal function of maturated smooth muscle cells is contraction regulating. However, smooth muscle cell can undergo phenotypic switching from differentiated stage to dedifferentiated stage, which characterized by reduced expression of smooth muscle specific genes, such as smooth muscle myosin heave chain, smooth muscle light chains, smooth muscle α-actin, SM22α, smooth muscle α-tropomyosin, smoothelin, h1-calponin, h-calponin, h-caldesmon, β-vinculin, metavinculin, telokin and desmin, whereas enhanced proliferation and migration^1,3^. Emerging evidence indicated that Traditional Chinese Medicine is effective in treatment variety cardiovascular diseases. Salvia miltiorrhiza is a traditional Chinese medicine and widely used for treatment of cardiovascular diseases. Tanshinone II A is a key ingredient separated from salvia miltiorrhiza. Previously reports demonstrated that Tanshinone II A attenuates angiotensin II induced cardiac hypertrophy and cardiac fibroblast proliferation through MEK/ERK pathway^4,5^. Tanshinone II A inhibits inflammatory response induced by myocardial infarction^6^. Tanshinone II A suppresses cells growth on human hepatocellular carcinoma cells^7^, human gastric carcinoma cells. Tanshinone II A prevents apoptosis in PC12 induced by serum starvation^8^. Tanshinone II A inhibits HCC cell invasion through suppressing the activity of MMPs^9^. In treatment of vascular diseases, Tanshinone II A attenuates atherosclerosis calcification through suppressing oxidative stress^10^. Tanshinone II A inhibits proliferation of pulmonary artery smooth muscle cells induced by hypoxia^11^. Tanshinone II A induces pulmonary artery smooth muscle cell apoptosis through suppressing JAK1/STATS signaling pathway^12^. And inhibits vascular smooth muscle migration through suppressing ERK1/2 MAPK signaling pathway^13^. However, whether Tanshinone II A plays a critical role during pathological vascular remodeling is largely unknown. Our preliminary data shown that Tanshinone II A significant promotes KLF4 expression. This study aimed to test hypothesis that Tanshinone II A regulates pathological vascular remodeling through KLF4 mediated smooth muscle cell phenotypic switching.

## Material and methods

### Mouse common carotid artery complete ligation injury animal model

Completed ligation of mouse left common carotid artery induced vascular remodeling was performed as previously described^14^. Briefly, Mouse were pretreated with Tanshinone II A (5 mg/kg) for 3 consecutive days and anesthetized with ketamine (80 mg/kg) and xylazine (5 mg/kg) by intraperitoneal injection^15^. Exposed the left common carotid arteries and completely ligated at bifurcation with 6-0 silk. The right common carotid artery was going the same process but not ligated. After Tanshinone II A consecutive treatment for 3 weeks and collected sections (5 µm) between 0.2 to 2.0 mm proximal to the ligation site. Morphological analysis based on HE-staining. The quantification of neointima areas and media layer area measured using Image J software.

### Rat aortic smooth muscle separation

Smooth muscle cells culture from thoracic artery of Sprague–Dawley rats separated as our previous report^16,17^. Briefly, the thoracic arteries for Sprague–Dawley rats harvested after euthanized and removed adhering periadventitial and endothelium under microscope. The adventitial layer removed after Blend enzyme III solution (Roche, 0.5 U/ml) digested for 5–15 min at 37 °C. The smooth muscle medial layer further digested with Blend enzyme III for 2 h at 37 °C. Collected the cells and suspended in DMEM medium contained 10% FBS.

### WST-1 cell proliferation assay

3 × 10^3^ rat smooth muscle cells (each well) were seeded in 96-well culture plate. Treated the cells with Tanshinone II A (1 μM) for 24 h^18^, and measured the absorbance at 450 nm using WST-1 proliferation assay.

### Scratch wound healing assay

Rat smooth muscle cells were seeded into 6-well culture plate with a density of 1 × 10^6^ cells/well. Treated the cells with Tanshinone II A (1 μM). A scratch across the center of the well gently and slowly made with a 10 µl pipette tip. The relative distance of the gaps monitored at different time points after crystal violet staining.

### Boyden chamber migration assay

Rat smooth muscle cells were treated with 1 μM Tanshinone II A for 24 h. Trypsinized and suspended 5 × 10^4^ cells in 100 μl serum-free medium, and seeded in Boyden chambers (353093, BD Biosciences, San Jose, CA, USA). Settle down the Boyden chambers into a 12-well culture plate, which contained 600 µl full culture growth medium contained 50 ng/ml Tanshinone II A and incubated at 37 °C for 4–24 h. The non-migrating cells on the upper side removed. The migrated cells on the bottom side fixed with 4% formaldehyde at room temperature for 20 min, and visualized after 0.1% Crystal violet staining. Migrated cells were counted manually in five random microscopic fields.

### BRDU incorporation assay

Rat smooth muscle cells treated with 1 μM Tanshinone II A overnight, following treatment with BRDU labeling reagent for 24 h. Fixed the cells with 4% PFA for 20 min at room temperature. Permeabilized with PBS contained 0.20% Triton-X-100 for 30 min, treated with 2 N HCl at room temperature for 30 min, blocked with 10% goat serum at room temperature for 1 h and incubated with BRDU antibody (Invitrogen).

### Quantitative real time PCR analysis

Total RNA from cells was extracted using TRIzol reagent. 600 ng RNA were using as template for reverse transcription with random hexamer primers using iScript cDNA synthesis kit^19^. Real time PCR performed duplicated on Bio-Rad real time PCR system with specific genes primers listed in table (Table 1). Relative gene expression level was analysis using the 2^−∆∆ct^ method against RPLPO.

**Table 1:** List of primer sequences used in the study primers used for quantitative RT-PCR.

Gene name	Species	Sequence
SM 22a	Rat	F: 5′-TGACATGTTCCAGACTGTTGACCTCT-3′
	Rat	R: 5′-CTTCATAAACCAGTTGGGATCTCCAC-3′
SM-α-actin	Rat	F: 5′-ATGCTCCCAGGGCTGTTTTCCCAT-3′
	Rat	R: 5′-GTGGTGCCAGATCTTTTCCATGTCG-3′
MHC	Rat	F: 5′-CAGTTGGACACTATGTCAGGGAAA-3′
	Rat	R: 5′-ATGGAGACAAATGCTAATCAGCC-3′
Calponin	Rat	F: 5′-AACTGGCACCAGCTGGAGAACATAG-3′
	Rat	R: 5′-GAGTAGACTGAACTTGTGTATGATTGG-3′
Myocardin	Rat	F: 5′-GTTCAGCTACCCTGGGATGCACCAA-3′
	Rat	R: 5′-GGCCTGGTTTGAGAGAAGAAACACC-3′
SRF	Rat	F: 5′-GATGGAGTTCATCGACAACAAGCTG-3′
	Rat	R: 5′-CCCTGTCAGCGTGGACAGCTCATA- 3′
CDKN1A	Rat	F: 5′-ATGACTGAGTATAAACTTGTGG-3′
	Rat	R: 5′-TCACATGACTATACACCTTGTC-3′
CDKN1B	Rat	F: 5′-GTCTCAGGCAAACTCTGAG-3′
	Rat	R: 5′-GTTTACGTCTGGCGTCGAAG-3′
CCND1	Rat	F: 5′-AATGGAACTGCTTCTGGTGAACA-3′
	Rat	R: 5′-CGGATGATCTGCTTGTTCTCATC-3′
pCNA	Rat	F: 5′-ACGTCTCCTTAGTGCAGCTTACTCT-3′
	Rat	R: 5′-TAATGATGTCTTCATTACCAGCACAT-3′
PTEN	Rat	F: 5′-GCACAAGAGGCCCTGGATT-3′
	Rat	R: 5′-TGAAACAACAGTGCCACTGG-3′
c-fos	Rat	F: 5′-GGGACAGCCTTTCCTACTACC-3′
	Rat	R: 5′-AGATCTGCGCAAAAGTCCTG-3′
Gadd45	Rat	F: 5′-ATGACTTTGGAGGAATTCTCGG-3′
	Rat	R: 5′-TCACCGTTCGGGGAGATTAATC-3′
KLF2	Rat	F: 5′-ACTTGCAGCTACACCAACTG-3′
	Rat	R: 5′-CTGTGACCCGTGTGCTTG-3′
KLF3	Rat	F: 5′-TCATGTACACCAGCCACCTG-3′
	Rat	R: 5′-TAGTCAGTCCTCTGTGGTTC-3′
KLF4	Rat	F: 5′-CGGGAAGGGAGAAGACACTGC-3′
	Rat	R: 5′-GCTAGCTGGGGAAGACGAGGA-3′
KLF5	Rat	F: 5′-AGCTCACCTGAGGACTCATA-3′
	Rat	R: 5′-GTGCGCAGTGCTCAGTTCT-3′
KLF15	Rat	F: 5′-GATGAGTTGTCACGGCACC-3′
	Rat	R: 5′-CACTGCGCTCAGTTGATGG-3′
RPLP0	Rat	F: 5′-GGACCCGAGAAGACCTCCTT-3′
	Rat	R: 5′-TGCTGCCGTTGTCAAACACC-3′

### Protein extraction and Western blotting

Total protein from rat smooth muscle cells lysed with RIPA buffer. Protein concentration determined using BCA kit. 20–40 μg protein from each groups denatured for SDS-PAGE. After blocked with 5% nonfat milk for 1 h, and incubated with specific antibodies at 4 ℃ for immunoblot analyses^19,20^.

### Hematoxylin and eosin (H&E) stain, immunohistochemistry (IHC) and immunofluorescence staining (IF)

The mouse carotid arteries fixed with 4% paraformaldehyde overnight at 4 °C and following paraffin embedded process, 5-μm thickness of slides collected and deparaffinized. Hematoxylin/eosin (H&E) staining performed as previously described^19,21^. For IHC staining, the deparaffinized slides were treated with citric acid and antigenic unmasked at 98 °C for 5 min, incubated with primary antibodies overnight at 4 °C, followed by incubation with biotinylated secondary antibody at room temperature for 1 h (Vector Laboratories, 1:200), and ABC solution (Vector Laboratories, Burlingame, CA, USA) for 30 min at room temperature. Expression level of the targets visualized after DAB solution incubation^20,22^. For IF staining for test BRDU incorporation, the deparaffinized slides, permeabilized with PBS contained 0.2% Triton-X-100, treated with 2 N HCl and blocked with 10% goat serum, incubated with primary antibodies overnight at 4 °C, washed with PBST and incubated with Alexa 594-conjugated secondary antibody at room temperature for 1 h. Nuclei visualized with 4′,6′-diamidino-2-phenylindole (DAPI) staining^17,19,21^. Images were collected using confocal microscopy (LS510, Zeiss).

### Statistics

Quantitative data presented as mean ± SEM. Comparisons between two groups were analysis by unpaired student’s *t* test using GraphPad prism software^19–21^. A value of P < 0.05 was considered statistically significant.

### Ethical approval

The use of mice and rat approved by the Experimental Animal Ethics Committee at Chengdu University of Traditional Chinese Medicine. Ethical approval number: 2019–04.

### Consent for publication

Yes.

## Results

### Tanshinone II A attenuates vascular injury induced neointimal hyperplasia

**Figure 1:**
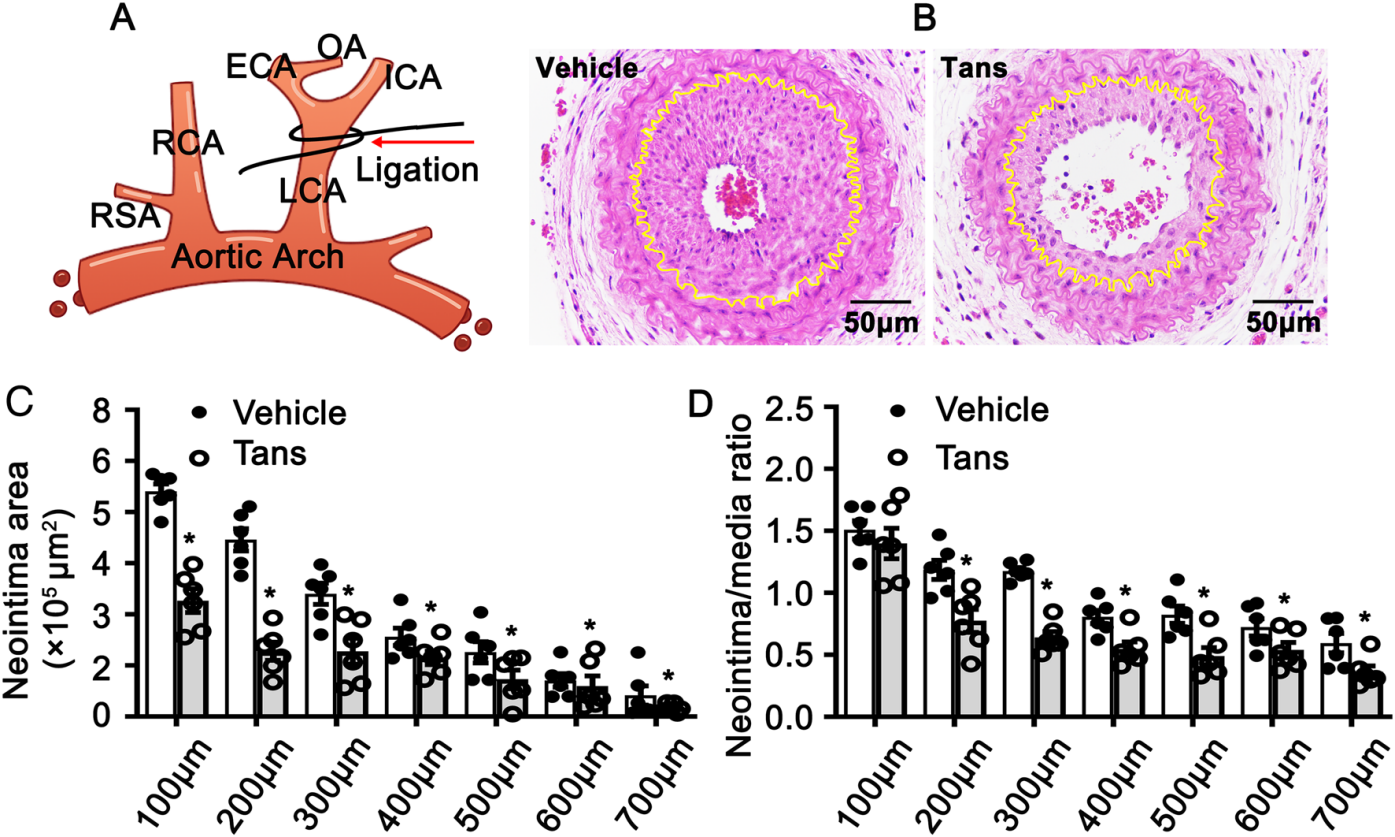
Tanshinone II A attenuates vascular neointimal hyperplasia in left common carotid artery ligated mice. (**A**) Schematic diagram for common left carotid artery ligation. (**B**) The representative images of H&E staining of the arteries. Mouse were pretreated with Tanshinone II A (5 mg/kg) for 3 consecutive days by intraperitoneal injection and following common left carotid artery ligation. After 3 consecutive weeks treatment with of Tanshinone II A, the arteries harvested and following paraffin embedded. (**C**) Neointimal area measured using Image J software (n = 6 mice per group). and the ratio of neointima area to the medium layer area shown in (**D**) (n = 6 mice per group). Data represented as mean ± SEM. *P < 0.05.

Tanshinone II A reported to suppress proliferation of smooth muscle cells. To determine whether Tanshinone II A plays a critical role in regulating smooth muscle cell phenotypic switching, we pretreated C57BL/6 mice with 5 mg/kg Tanshinone II A by intraperitoneal injection for 3 consecutive days and following left common carotid artery ligation to induce vascular injury (Fig. 1A). Following three weeks of consecutive treatment with Tanshinone II A, harvested the arteries and undergoing paraffin embedded. We performed H&E staining to visualize vascular morphological change induced by vascular injury. Our results indicated that treatment with Tanshinone II A dramatically suppresses neointima formation (Fig. 1B). We analyzed neointima areas using Image J software from different locations far away from the ligation site. Our data shown the neointima areas from 100 to 700 μm were significant decreased (Fig. 1C). We compared the ratios of neointima areas to the medium layer areas, which were significant decreased (Fig. 1D). Those data indicated that Tanshinone II A involves in regulating vascular remodeling induced by vascular injury.

### Tanshinone II A regulates smooth muscle phenotypic switching

**Figure 2:**
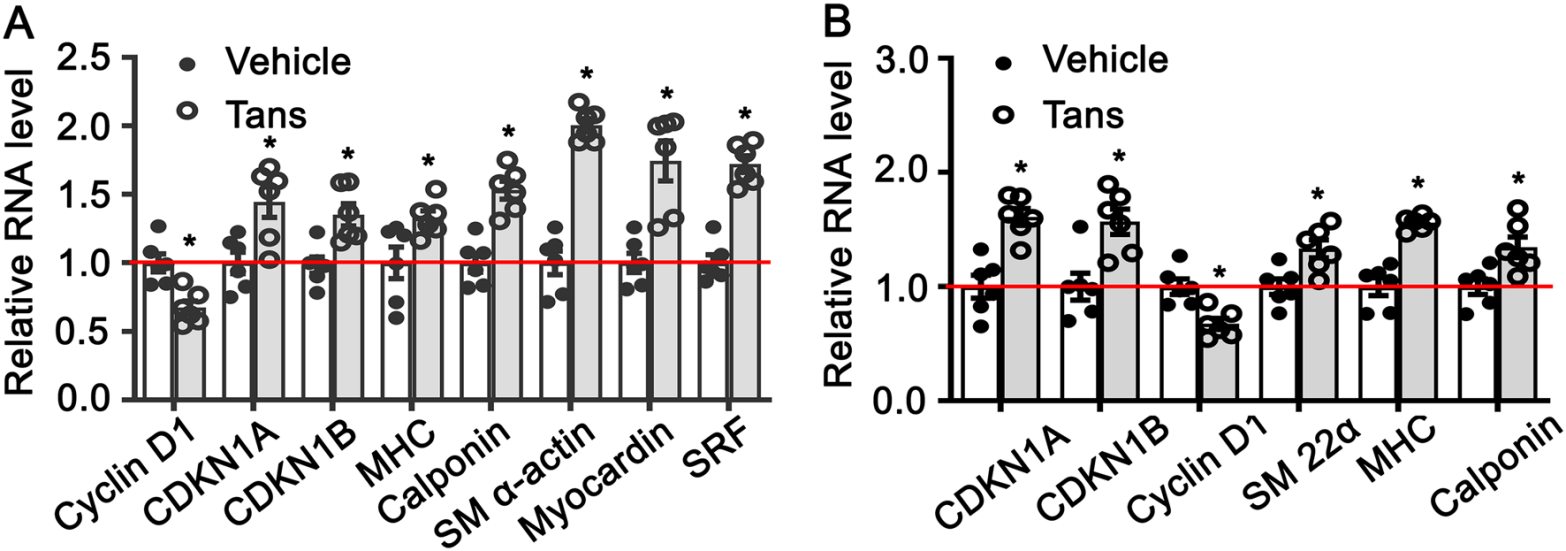
Tanshinone II A regulates rat aortic smooth muscle cell phenotypic switching. (**A**) Rat SMCs were treated with Tanshinone II A (1 μM) for 36 h and real time PCR performed to detect expression of cell growth related genes, Cyclin D1, CDKN1A, CDKN1B and smooth muscle specific genes (n = 6 independent experiments). (**B**) Growth of rat smooth muscle cells induced by PDGF-BB treatment (20 ng/mL) and real time PCR performed to observe the expression of smooth muscle differentiated genes and cell growth related genes (n = 6 independent experiments). Data represented presented as mean ± SEM. *P < 0.05.

Smooth muscle cells phenotypic switching is critical for Pathological vascular remodeling. To determine whether Tanshinone II A contributes to smooth muscle cell phenotypic switching in vitro, we treated rat aortic smooth muscle cells with tanshinone IIA (1 μM) for 30 h, and real time PCR performed to evaluate the expression of SMC differentiated genes and cell growth-regulating genes. Our data indicated that Tanshinone II A treatment significant promotes expression of smooth muscle specific genes, including MHC, calponin, SM α-actin, myocardin and SRF, whereas dramatically suppresses Cyclin D1 expression (Fig. 2A). We further treated rat smooth muscle cell with PDGF-BB to induce cell growth (Supplementary Fig. 1A,B). The data shown that tanshinone II A treatment attenuates PDGF-BB induced cell growth and expression of Cyclin D1, whereas enhances the expression of MHC, Calponin, SM 22α, myocardin (Fig. 2B). We next induced rat smooth muscle cell differentiation by rapamycin treatment (Supplementary Fig. 2A,B). Tanshinone II A treatment promotes the expression of smooth muscle differentiated marker genes, including SM 22α, MHC, Calponin, myocardin (Supplementary Fig. 3). Those data suggested that Tanshinone II A modulates smooth muscle phenotypic switching.

### Tanshinone II A suppresses migration of smooth muscle cells

**Figure 3:**
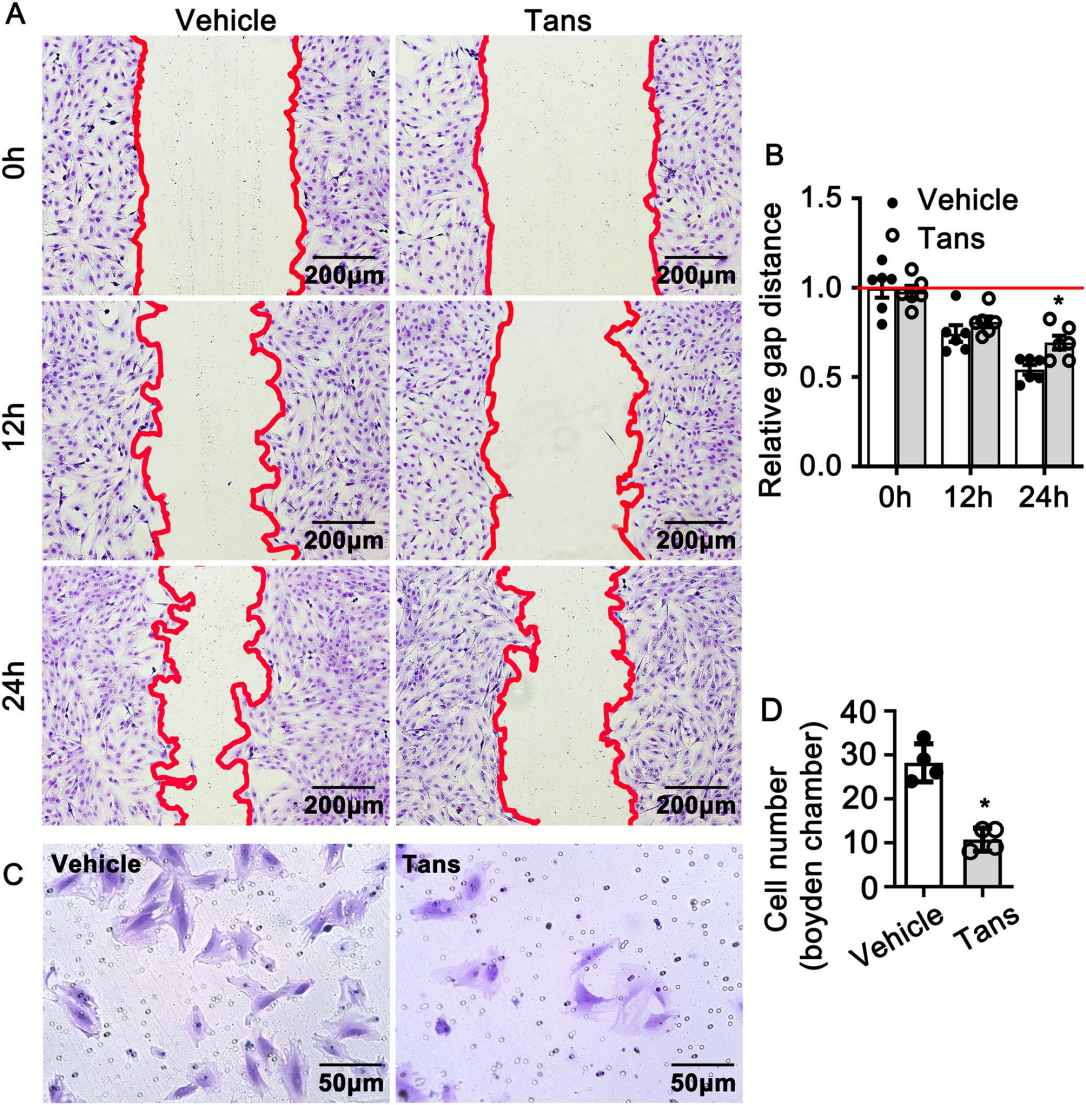
Tanshinone II A suppresses rat aortic smooth muscle cell migration. (**A**) Rat smooth muscle cells treated with 1 μM Tanshinone II A for 24 h and Wound scratch experiment performed. The relative gap distance shown in (**B**) (n = 6 independent experiments). (**C**) Rat smooth muscle cells treated with Tanshinone II A (1 μM) and Boyden Chamber Migration Assay was performed, the quantification of migrated cells were exhibited in (**D**) (n = 4 independent experiments). Data represented means ± SEM. *P < 0.05.

Migration of VSMCs contributes to neointimal hyperplasia after vascular injury^23^. To determine the function role of Tanshinone II A in regulating smooth muscle cell migration, we treated rat smooth muscle cells with Tanshinone II A (1 μΜ) and wound scratch healing assay was performed to monitor the scratching gap at different time point. No difference exhibited at 12 h. However, the distance of scratching gap is larger after Tanshinone II A treatment at 24 h (Fig. 3A,B). Similar results were obtained using Boyden chamber migration assay (Fig. 3C,D). Those data demonstrated that Tanshinone II A treatment dramatically suppresses migration of rat aortic smooth muscle cells.

### Tanshinone II A suppresses proliferation of smooth muscle cells

**Figure 4:**
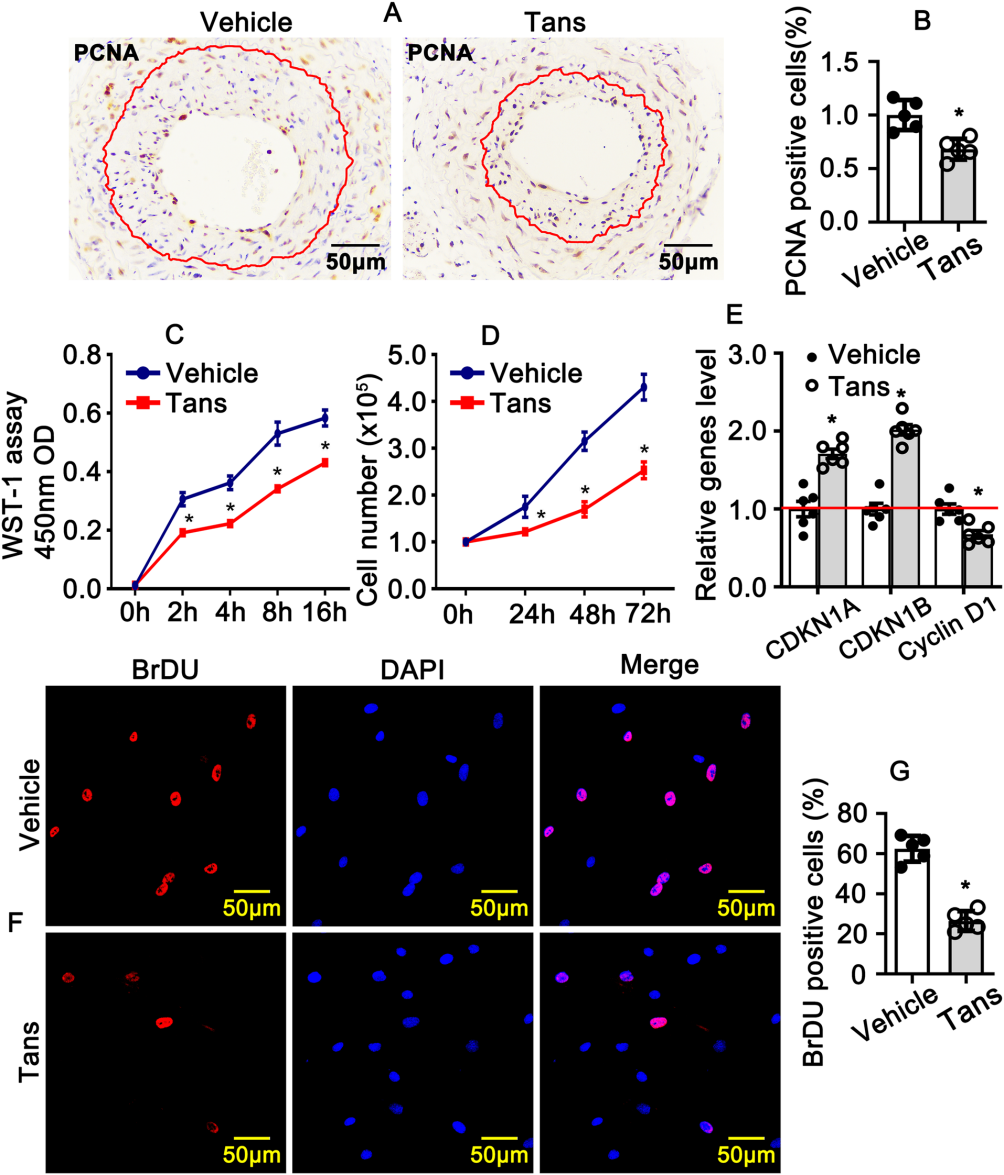
Tanshinone II A suppresses rat aortic smooth muscle cells proliferation. (**A**) Representative Images of IHC staining from left common carotid artery complete ligation injury animal model against with PCNA antibody. (**B**) Quantification of PCNA positive smooth muscle cells in the neointima areas from (**A**) (n = 5 mice per group). (**C**) Rat smooth muscle cells treated with 1 μM Tanshinone II A for 24 h and proliferation and viability were detected by WST-1 assay (n = 5 independent experiments). (**D**) Rat smooth muscle cells treated with 1 μM Tanshinone II A and cell number counted at different time point (n = 5 independent experiments). (**E**) Tanshinone II A treated rat smooth muscle cells for 30 h and real time PCR performed to investigate the expression of cell cycles related genes (n = 6 independent experiments). (**F**) Representative images of IHC staining against BRDU antibody from rat smooth muscle cells. Rat smooth muscle cells treated with 1 μM Tanshinone II A overnight, following the treatment with BRDU labeling reagent for 24 h and following IHC staining, the nuclei visualized using DAPI staining. The BRDU positive cells from (**F**) quantified in (**G**) (n = 5 independent experiments). The quantify data represented as means ± SEM. *P < 0.05.

Smooth muscle cell phenotypic switching is characterized extremely reduced expression of differentiated markers genes and enhanced proliferation. To determine the function role of Tanshinone II A in regulation smooth muscle cell proliferation, immunohistochemistry staining against PCNA was performed on slides from completed ligation of mouse left common carotid arteries (Fig. 4A). The percentage of PCNA positive smooth muscle cells within neointima area was extensive decreased (Fig. 4B). We next sought to determine whether Tanshinone II A treatment could suppress rat aortic smooth muscle cells growth. After Tanshinone II A treatment, we monitored the proliferation and viability of smooth muscle cells using WST-1 assay. The results indicated that absorbance (OD) at 450 nm is remarkable decreased after transhinone II A treatment (Fig. 4C). Seminar results were obtained from cell number counting experiment, Tanshinone II A treatment extremely decreases the cell numbers (Fig. 4D). Our Real time PCR data shown that Tanshinone II A treatment suppresses Cyclin D1 expression and promotes expression of cell cycle negative regulated genes, CDKN1A and CDKN1B (Fig. 4E). We further confirmed the results by BRDU incorporation assay. We treated rat smooth muscle cells with Tanshinone II A and following treated with BRDU labeling reagent, immunofluorescent staing was performed against BRDU antibody, the percentage of BRDU positive cell was quantified. Our data shown that Tanshinone II A treatment dramatically suppressed BRDU incorporation (Fig. 4F,G). These results above demonstrated that Tanshinone II A treatment significant suppresses rat aortic smooth muscle cell proliferation.

### Tanshinone II A promotes KLF4 expression during smooth muscle phenotypic switching

**Figure 5:**
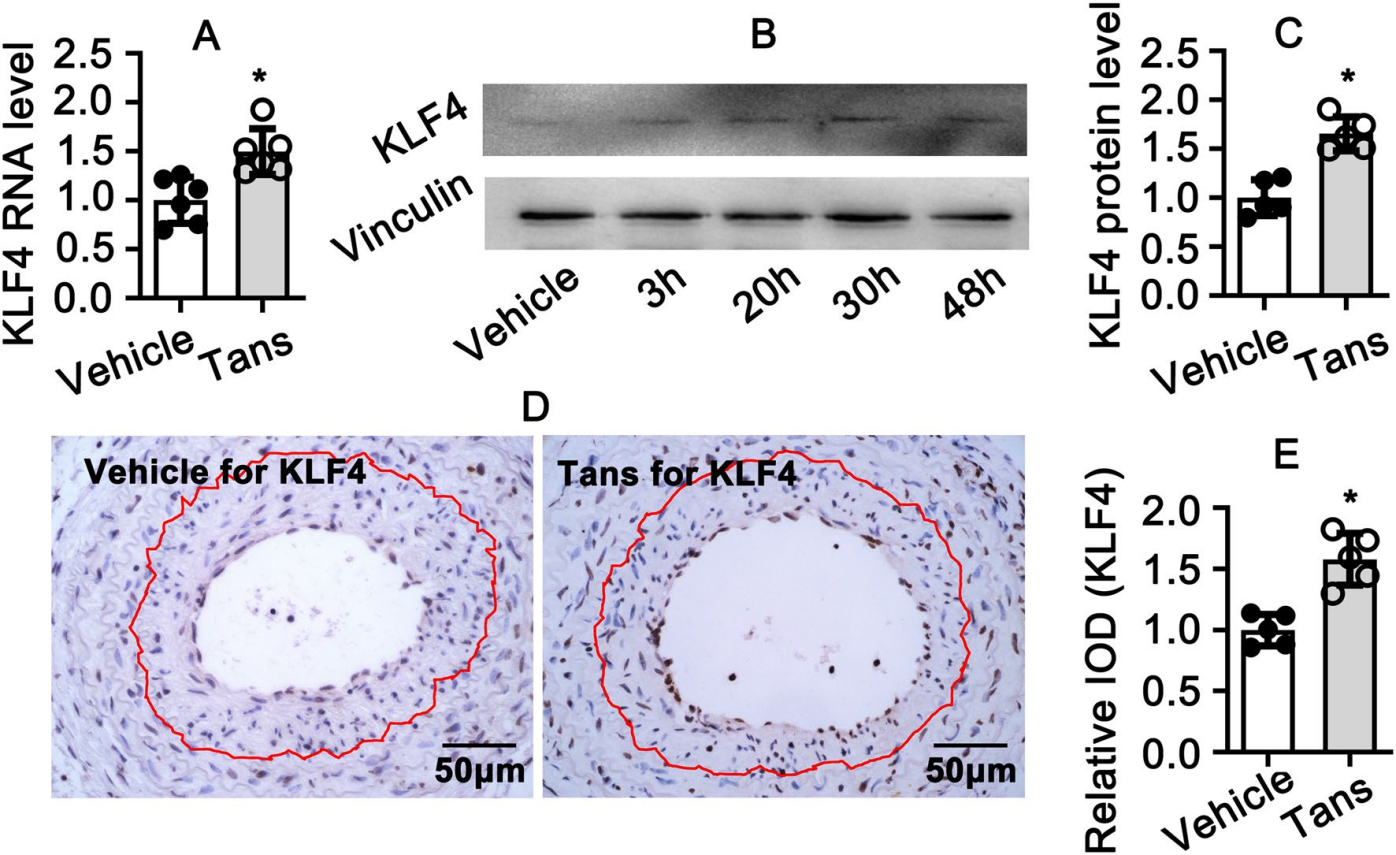
Tanshinone II A treatment promotes KLF4 expression. (**A**) Rat smooth muscle cells treated with Tanshinone II A (1 μM) for 30 h and real time PCR performed to detect KLF4 transcription level (n = 6 independent experiments). (**B**) Rat smooth muscle cells treated with Tanshinone II A (1 μM) for 3 h, 20 h and 30 h. The expression of KLF4 investigated by western blotting and the quantification data showed in (**C**) (n = 5 independent experiments). (**D**) Representative Images of IHC staining from left common carotid artery complete ligation injury animal model against with KLF4 antibody. (**E**) Relative expression level of KLF4 in (**D**) was quantified by integrated optical density (IOD) using Image J software (n = 5 mice per group). Data represented as means ± SEM. *P < 0.05.

Tanshinone II A plays a critical role in regulating smooth muscle cell phenotypic switching. However, the underlie mechanism is poorly understood. We used rat aortic smooth muscle cells and treated with 1 μM Tanshinone II A for 30 h, real time PCR performed to screen multiple signaling pathways. Interesting, we observed that Tanshinone II A treatment promotes the expression of Pten, c-Fos, Gadd45, KLF4 and KLF1 expression (Supplementary Fig. 4). Since KLF4 plays an pivotal role during smooth muscle cell phenotypic switching^24^, we confirmed the expression of KLF4 after Tanshinone II A treatment. Our data demonstrated that Tanshinone II A treatment obviously enhances the expression level of KLF4 at both transcription level and protein level (Fig. 5A–C). This was consistent with our in vitro study. We performed immunohistochemistry staining of KLF4 in common carotid arteries. The expression of KLF4 within neointima area dramatically decreased after Tanshinone II A treatment (Fig. 5D,E). Our results suggested that Tanshinone II A promotes KLF4 expression in rat aortic smooth muscle cell.

### Interruption KLF4 expression contributes to Tanshinone II A in regulating pathological vascular remodeling

**Figure 6:**
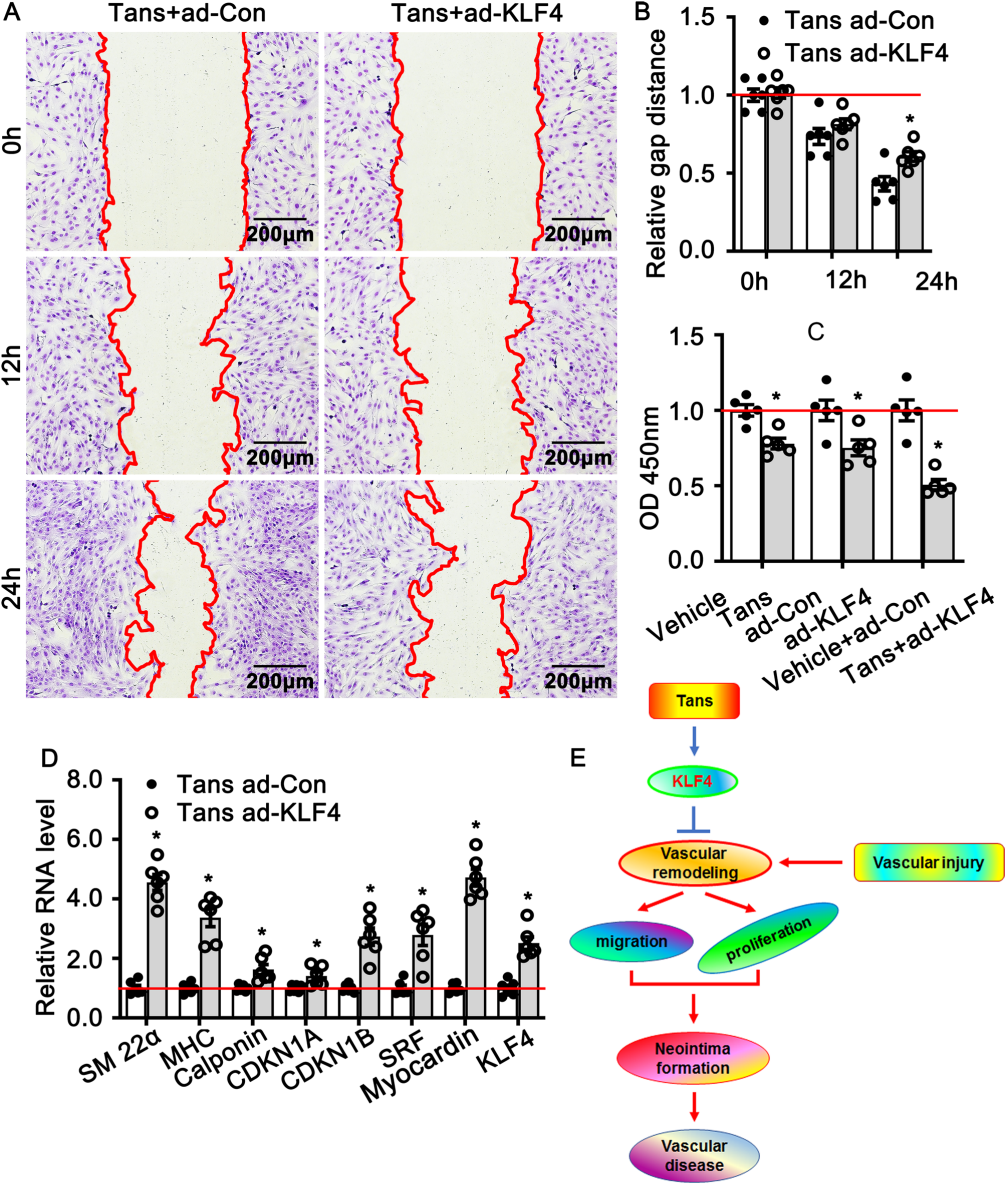
Tanshinone II A regulates smooth muscle phenotypic switching through enhances KLF4 expression. (**A**) Wound healing assay was performed to detect migration of KLF4 overexpress VSMCs treated with Tanshinone II A in 3 different time periods (0 h, 12 h, 24 h). The quantification of relative gap distance from (**A**) displayed in (**B**) (n = 6 independent experiments). (**C**) WST-1 proliferation assay was used to assess proliferation of rat smooth muscle cells with different treatment (n = 5 independent experiments). (**D**) Real Time PCR performed to detect transcription level of smooth muscle cell phenotypic switching regulating genes (n = 6 independent experiments). (**E**) Schematic diagram indicates that Tanshinone II A attenuates vascular remodeling through klf4 mediated smooth muscle cell phenotypic switching.

To determine whether Tanshinone II A regulates smooth muscle cell phenotypic switching through mediated KLF4 expression, we first overexpressed of KLF4 in rat aortic smooth muscle cells by adenovirus infection and the expression efficiency validated by real time PCR (Supplementary Fig. 5). We next sought to determine whether overexpression of KLF4 in smooth muscle cell could interrupt Tanshinone II A in regulating smooth muscle cell migration. We infected rat aortic smooth muscle cells with adenoviral KLF4 to generate KLF4 overexpression smooth muscle cells, and treated with 1 μM Tanshinone II A. The migration was monitored scratch wound healing assay. Our results indicated that overexpression of KLF4 suppresses migration of rat aortic smooth muscle cells (Fig. 6A,B). We further detected whether overexpression of KLF4 exacerbates Tanshinone II A in suppression smooth muscle cell proliferation using WST-1 cell proliferation assay. Both Tanshinone II A treatment and adenovirus mediated KLF4 overexpression can inhibit rat aortic smooth muscle cell proliferation. However, after adenovirus infection and following Tanshinone II A treatment, the cells exhibited much lower OD compared to the control group (Fig. 6C). We finally determined whether overexpression of KLF4 interrupts Tanshinone II A in regulating the expression of smooth muscle differentiated marker genes using real time PCR. Overexpression of KLF4 in rat aortic smooth muscle cells promotes the expression of SM 22α, MHC, calponin, SRF and Myocardin (Fig. 6D).

Taken together, our data in this study demonstrated that Tanshinone II A attenuates smooth muscle cell phenotypic switching induced by vascular injure partially through regulating KLF4 expression (Fig. 6E).

## Discussion

This study for the first time provides the evidence that Tanshinone II A administration suppresses vascular remodeling through KLF4 mediated smooth muscle cell phenotypic switching. We found that Tanshinone II A intraperitoneal injection attenuates complete carotid artery ligation induced neointima formation. Tanshinone II A attenuates rat aortic smooth muscle cell growth induced by PDGF-BB treatment, and promotes smooth muscle cell differentiated marker genes expression induced by Rapamycin. The critical role of Tanshinone II A in regulating smooth muscle cell phenotypic switching exhibited in dramatically promoting the expression of smooth muscle cell differentiated marker genes, as well as impaired cell proliferation.

The Traditional Chinses Medicine, salvia miltiorrhiza, is traditional used in treatment of cardiovascular diseases. At least 108 kinds of different integrants have been identified from salvia miltiorrhiza. However, the biofunction of most integrants are undefined. In our study, we only focused on a key integrant, Tanshinone II A, and observed its function in regulating smooth muscle cell phenotypic switching.

In this study we reported a valuable mechanism that Tanshinone II A regulates smooth muscle phenotypic switching through enhanced KLF4 expression to suppress smooth muscle cells proliferation, whereas induce differentiate, and eventually regulates pathological vascular remodeling.

KLF4, a member of Krüppel-like family of transcription factors (KLFs), plays a crucial role in various vascular diseases. Expression level of KFL4 is extremely low in normal condition, whereas the expression level dramatically increased in response to injury stresses. We observed that Tanshinone II A treatment promotes the expression of Pten, c-Fos, Gadd45 and Krüppel-like family of transcription family (KLFs). We detected the other members of Krüppel-like family of transcription family, including KLF2, KLF3, KLF5 and KLF15, the expression of KLF4 is remarkable increased compared to the other members (Supplementary Fig. 3). We tried to knockout KLF4 in rat smooth muscle cells using adenovirus infection. However, the knockdown efficiency of KLF4 virus was not high enough. While forced expression of KLF4 in rat smooth muscle cell aggravated the function of Tanshinone II A in suppressing proliferation and promoting expression of muscle differentiated genes (Fig. 6). Those results suggested Tanshinone II A regulates smooth muscle phenotypic switching, at least partially, through active KLF4 signaling pathway.

In this study we did not adopted multiple biological methods to detect the interaction between Tanshinone II A and KLF4, as endogenously genes which involved in signaling pathway transmission.

Tanshinone II A treatment suppresses rat aortic smooth muscle growth. Our real time PCR data indicated that Tanshinone II A treatment dramatically enhances the expression of CDKN1A and CDKN1B, which are cell cycle negative control genes. The results suggested that Tanshinone II A, at least partially, inhibits cell cycle to regulate cell growth.

Rapamycin induced smooth muscle cell differentiation^25^. After Rapamycin and Tanshinone II A treatment, our real time PCR data shown that Tanshinone II A treatment can not significant promote smooth muscle differentiated genes expression (Supplementary Fig. 3). The optimal explanation is that both control and Tanshinone II A treatment smooth muscle cell have been treated with Rapamycin. We treated smooth muscle cell with Tanshinone II A alone dramatically promotes differentiated genes expression (Fig. 2).

In summary, our study not only demonstrate that Tanshinone II A is critical in regulating smooth muscle cell pathological phenotypic switching, but also identify a target for vascular diseases treatment.

## Conclusion

Tanshinone II A plays a pivotal role in regulating pathological vascular remodeling through KLF4 mediated smooth muscle cell phenotypic switching. This study demonstrated that Tanshinone II A is a potential therapeutic agent for vascular diseases.

## Supplementary information

Supplementary Legends.

Supplementary Figure 1.

Supplementary Figure 2.

Supplementary Figure 3.

Supplementary Figure 4.

Supplementary Figure 5.

## Data Availability

The authors state that all relevant data are available within the article and the online Supplementary material or are available from the corresponding authors upon reasonable request.

## Abbreviations

Tans: Tanshinone II A
KLF4: Krüppel-like factor 4
HCC: Hepatic cell carcinoma
PDGF-BB: Platelet-derived growth factor BB
PCR: Polymerase chain reaction
ERK: Extracellular signal regulated protein kinase
JAK1: Janus kinase 1
STAT1: Signal transduction and activation of transcription-1
H&E: Hematoxylin and eosin stain
IHC: Immunohistochemistry
IF: Immunofluorescence staining
BRDU: 5-Bromo-2-deoxyUridine
DAPI: 4′,6′-Diamidino-2-phenylindole
PCNA: Proliferating cell nuclear antigen
SM22α: Smooth muscle protein 22α
MHC: Myosin heavy chain
