# Supplementary material for: Tanshinone II A attenuates vascular remodeling through klf4 mediated smooth muscle cell phenotypic switching
Source: Sci Rep. 2020 Aug 17;10:13858. doi: 10.1038/s41598-020-70887-1 (PMC7431534; doi:10.1038/s41598-020-70887-1)
Supplement: Supplementary file 1 — Supplementary Legends. [file 41598_2020_70887_MOESM1_ESM.pdf]

Tanshinone II A attenuates vascular remodeling through klf4 mediated smooth muscle cell phenotypic switching

Guanhua Lou [a, b], Wangming Hu [a], Ziqiang Wu [b], Huan Xu [a], Huan Yao [a], Yang Wang [a], Qinwan Huang [a], Baojia Wang [a], Li Wen [a], Xiongbing Chen [a], Yaping Shi [a], Lan Yang [a], Yiming Xu [c], Yong Wang [a]

[a] Chengdu University of Traditional Chinese Medicine, College of Basic Medicine, Chengdu, China.

[b] Chengdu University of Traditional Chinese Medicine, College Pharmacy, Chengdu, China.

[c] Guangzhou Medical University, School of Basic Medical Sciences, Guangzhou, China.

Running title: **Tanshinone II A regulates smooth muscle cell phenotypic switching**

Corresponding author: Yong Wang, PhD.

Basic Medical College,

Cheng Du University of Traditional Chinese Medicine,

Chengdu, Sichuan, China.

Email: yongwang1008@hotmail.com

Supplementary Figure legends

Supplementary Fig 1. PDGF-BB induced proliferation of rat aortic smooth muscle cells. A. Rat smooth muscle cells treated by PDGF-BB treatment (20ng/mL), cell growth and viability measured by WST-1 assay (n=5 independent experiments). B. Real time PCR performed to detect the expression of cell growth related genes (n=6 independent experiments). Data represented as means ± SEM. * $P < 0.05$.

Supplementary Fig 2. Rapamycin induced differentiation of rat aortic smooth muscle cells. A. Differentiation of rat smooth muscle cells induced by Rapamycin (1μM) treatment, cell growth and viability monitored by WST-1 assay (n=5 independent experiments). B. Real time PCR performed to investigate the expression of smooth muscle specific genes after rapamycin treatment (n=6 independent experiments). Data presented as mean ± SEM. Experiments repeated twice. $P < 0.05$.

Supplementary Fig 3. Tanshinone II A attenuates Rapamycin induced smooth muscle differentiation. Rat smooth muscle cell treated with Tanshinone II A (1μM) overnight and following Rapamycin treatment for 30 hours, and real time PCR performed to investigate the expression of smooth muscle differentiated genes (n=6 independent experiments). Data presented as mean ± SEM.

Supplementary Fig 4. Rat smooth muscle cell treated with Tanshinone II A (1μM) for 30 hours and real time PCR performed to investigate the expression of genes which play a critical in regulating smooth muscle cell phenotypic switching (n=6 independent experiments). Data presented as mean ± SEM. $P < 0.05$.

Supplementary Fig 5. Overexpression of KLF4 in rat smooth muscle cells induced by

adenovirus infection and Real Time PCR performed to validate the overexpression efficiency (n=6 independent experiments). Data presented as mean ± SEM. $P < 0.05$.
